# Perspectives From French and Filipino Parents on the Adaptation of Child Health Knowledge Translation Tools: Qualitative Exploration

**DOI:** 10.2196/33156

**Published:** 2022-03-25

**Authors:** Sarah A Elliott, Kelsey S Wright, Shannon D Scott, Lisa Hartling

**Affiliations:** 1 Alberta Research Centre for Health Evidence Department of Pediatrics University of Alberta Edmonton, AB Canada; 2 Cochrane Child Health Department of Pediatrics University of Alberta Edmonton, AB Canada; 3 Faculty of Nursing University of Alberta Edmonton, AB Canada

**Keywords:** knowledge translation, cultural adaptation, health evidence, dissemination, child health

## Abstract

**Background:**

A number of evidence-based knowledge translation (KT) tools for parents of children with acute health conditions have been developed. These tools were created and tested with parental input and disseminated to groups proficient in English. Therefore, it is unclear whether they are useful for populations that are more diverse. To enhance the reach of our current and future KT tools, language translation and cultural adaptations may promote relevance for previously underserved knowledge users.

**Objective:**

This study aims to explore and understand considerations for the cultural and linguistic adaptation of a KT tool in French and Filipino communities.

**Methods:**

A KT tool (whiteboard animation video) describing the signs and symptoms of croup was originally developed in English to provide parents with evidence-based information couched within a narrative reflecting parents’ experiences with the condition. This KT tool was adapted (linguistics and imagery) for French- and Tagalog-speaking parents and caregivers through feedback from key stakeholders. The videos were presented to the respective language speakers for usability testing and discussion. Participants were asked to view the KT tool, complete a usability survey, and participate in semistructured interviews. Audio recordings from the interviews were transcribed verbatim, translated into English, and analyzed for relevant themes by using thematic analysis.

**Results:**

French- (n=13) and Tagalog-speaking (n=13) parents completed the usability survey and were interviewed. Although analyzed separately, both data sets produced similar findings, with key themes relating to understanding, relatability, and accessibility. Both the French and Tagalog groups reported that the video and other KT tools were useful in their adapted forms. Participants in both groups cautioned against using verbatim vocabulary and suggested that cultural competency and understanding of health languages were essential for high-quality translations. Parents also discussed their preference for videos with diverse visual representations of families, home environments, and health care workers, as such videos represent their communities more broadly.

**Conclusions:**

French and Filipino parents appreciated having KT tools in their first language; however, they were also supportive of the use of English KT products. Their suggestions for improving the relatability and communication of health messages are important considerations for the development and adaptation of future KT products. Understanding the needs of the intended end users is a crucial first step in producing relevant tools for health evidence dissemination.

## Introduction

### Background

In the context of pediatric health care, connecting parents and caregivers to research evidence has the power to improve health decision-making and access to support services [1-3]. Knowledge translation (KT) facilitates the actioning of evidence to improve such outcomes. Furthermore, by integrating patient experiences, KT tools (eg, videos, e-books, and infographics) may be more relevant and impactful [4].

Parents and caregivers have become increasingly reliant on web-based sources for health information [5,6]. Subsequently, we developed >25 digital KT tools to improve dissemination and reach of health information for parents seeking help for acute childhood conditions (eg, bronchiolitis, croup, acute otitis media, and fever) [7-11]. The theoretical foundation underpinning our work was the Knowledge to Action framework [12], and the process development for our tools is situated within the tailoring *knowledge* portion of the knowledge to action cycle. Our research teams worked with clinicians and families to co-develop and disseminate the KT tools for Albertans and Canadians in general [13]. Throughout the process, our team engaged with and integrated end user (parent) feedback to ensure that our KT tools were devised and tested with the health consumer in mind [13]. However, our end users have often represented majority cultures in our community, and our KT tools were piloted with and disseminated primarily to English-speaking residents.

Although integrating feedback from end users or stakeholders has become a standard practice for many involved in KT tool development, those providing feedback commonly represent majority voices in the health care community [14,15]. Few studies have explored the usability of KT tools with minority cultural groups [16], and even fewer have tested culturally adapted tools with their target populations [17].

### Objective

Our goal was to broaden the reach of our work to different linguistic and cultural contexts to enhance knowledge and awareness among diverse user groups. The objective of this study is to explore how to adapt these tools to 2 non–English-speaking groups. Alberta is a diverse province and continues to grow in represented cultures. Other than English, French and Tagalog are the most common languages spoken in Alberta homes [18]. The relatively high proportion of those who speak French and Tagalog prompted our decision to include these communities in our efforts to understand cultural adaptations for consideration in KT tool development.

To address the different cultural communities in Alberta, we adapted a pre-existing KT tool for French- and Tagalog-speaking parents. By engaging community members, we aim to explore the key cultural aspects of French and Filipino parent experiences as well as to understand considerations for cultural adaptation processes in general.

## Methods

### The KT Tool

A pre-existing English digital whiteboard animation video depicting the signs and symptoms of croup was chosen for adaptation [18]. This topic was chosen for adaptation as croup is one of the most common causes of upper airway obstruction in children and accounts for significant rates of emergency department visits in Canada [19]. This video was originally produced through consultation with predominantly White, English-speaking parents [20]. Recent usability testing showed that parents rated the tool highly and gave favorable scores to all usability questions. Parents also reported that the video was informative, easy to understand, short and to the point, and visually appealing [21]. Audio and visuals of the original video were representative of mainstream Canadian health care providers’ and parents’ experiences.

### Adaptation Process

#### Overview

Although the adaptation process for the 2 different cultural groups varied slightly, in the absence of a cultural adaptation theory, model, or framework for KT tools (or KT interventions), we used elements of the Ecological Validity Framework by Bernal et al [22]. Adaptations were considered to be related to language (translation and differences in regional or subcultural groups), persons (patient–health care provider relationship, family roles), metaphors (symbols and sayings), and content (values, customs, and traditions).

#### French Adaptation Process

The adaptation process for French-speaking parents was purely linguistic. French-speaking members of the research team translated the original English video script into French by using a forward**-**back translation process [23]. French-speaking researchers and clinicians were consulted during the script translation process to ensure appropriate communication of the medical terminology. A video production company then incorporated the linguistic changes by creating a new French narration and editing visual text to French terminology (Multimedia Appendix 1). Once edits from the digital media company were approved by key stakeholders, the tool was ready for evaluation by the end user group (ie, French-speaking parents).

#### Tagalog Adaptation Process

The adaptation process for Tagalog-speaking parents involved linguistic and visual adaptation. The linguistic adaptation component of this process was similar to the French adaptation, with narration and video text translation by Tagalog-speaking members of the research team, integration by the digital media company, and review by key stakeholders. We also adapted character appearances to more similarly represent Filipino community members. Through continual consultation with Filipino parent stakeholders and graphic designers from the digital media company, character visuals were adapted to represent the Filipino community more accurately (Multimedia Appendix 1). Linguistic and visual edits from the digital media company were approved by key stakeholders before evaluation.

### Participants and Ethics Approval

Participants were purposively sampled based on self-reported preference for speaking French or Tagalog at home, having English as a second language, and having a parent or guardian role of a child aged <18 years. Ethics approval was granted by the University of Alberta Health Research Ethics Board (Pro00087285 and Pro00085766). All the study documents were translated into French and Tagalog (recruitment materials, study information letters, consent forms, usability surveys, and interview guides). All participants provided informed consent before data collection. Recruitment materials were posted on social media platforms and bulletin boards throughout the respective communities.

### Usability Survey

In both studies, parents were asked to complete a usability survey in French or Tagalog after viewing the adapted KT tool. The survey (Multimedia Appendix 2) collected demographic information and evaluated the video’s quality of information, format, appropriateness of visuals, and communication of health information. The survey content was informed by a knowledge synthesis of over 180 usability evaluations [24]. Participants were instructed to state their agreement with 9 statements (eg, “it is simple to use” and “its length is appropriate”) on a 5-point Likert scale ranging from *strongly disagree* to *strongly agree*. Following survey completion, parents were invited to participate in one-on-one semistructured interviews to elaborate on their survey responses and speak about the usability of the translated tool within their communities.

### Interviews

French- and Tagalog-speaking research team members conducted interviews with French and Filipino parents who completed the usability survey. These interviews occurred in French or Tagalog with research team members who were trained in qualitative data collection, following an interview guide and asking relevant probing questions. The interview questions were chosen to explore the participants’ perspectives on general cultural considerations, as well as specific feedback for the adapted croup video (Multimedia Appendix 3). The interviews were recorded, transcribed verbatim by a native French- or Tagalog-speaking translator, and then translated into English for analysis. Conducting the interviews in French and Tagalog allowed participants to communicate their perspectives in their preferred language [25]. The decision to translate interview transcripts was appropriate for communicating findings within the predominantly English research team and readership audience. This choice was methodologically consistent for thematic analysis, unlike more deeply phenomenological methodologies in which the participant language could articulate nuances in experience [26]. Nevertheless, any cross-language qualitative study can introduce concerns regarding data trustworthiness [27]. To mitigate potential translation errors, the bilingual research team members validated the translated transcripts and communicated with the broader research team. The English field notes taken by the interviewers also aided in the cross-language study process, where participants’ nonverbal responses were noted.

### Analysis

Descriptive statistics were used to describe the study sample and the usability survey results. Interview data collection and analysis occurred concurrently until no new responses transpired. The translated transcripts were analyzed using inductive thematic analysis [28]. Data management and analysis were facilitated using NVivo 12 software (version 12; QSR International). The analysis process was iterative, where each transcript was read in its entirety, verbatim codes were assigned to topics in the transcript, and codes were grouped into preliminary categories. The preliminary categories from all the coded transcripts were compared and organized into themes. Common themes were reviewed and verified by all authors. Interviewers wrote field notes during the interviews to promote confirmability, reflexive practice, and rigorous research processes [29]. The trustworthiness of the data was guided by three criteria: fairness, ontological and educative authenticity, and catalytic and tactical authenticities [29]. Fairness was addressed through detailed field notes, interview recordings, and transcripts. Ontological and educative authenticity were addressed through an inductive interview process in which participant perspectives were considered true. Catalytic and tactical authenticities were addressed through continual consultation with key stakeholders, positioning the participants as experts of their own experiences. Analytic rigor was promoted through communication within the research team and the verification of themes. Bilingual research team members validated translated interview transcripts to mitigate interpretation errors that can occur in cross-language qualitative research [27].

## Results

### Overview

A total of 13 French- and 13 Tagalog-speaking parents residing in Alberta completed the study. The participant demographics are shown in Table 1. Briefly, most French-speaking participants were mothers (12/13, 92%) and were born in Canada (8/13, 62%), whereas Tagalog-speaking participants were mothers (9/13, 69%) and fathers (4/13, 31%) born outside Canada (12/13, 92%).

**Table 1:** Demographic characteristics of French and Filipino parents who participated in usability testing and interviews (N=26).

Variable		Participants, n (%)	
		French (n=13)	Filipino (n=13)
**Parenting role**			
	Father	1 (8)	4 (31)
	Mother	12 (92)	9 (69)
**Age (years)**			
	20-30	2 (15)	1 (8)
	31-40	6 (46)	6 (46)
	41-50	4 (31)	5 (38)
	≥51	1 (8)	1 (8)
**Marital status**			
	Married	10 (77)	13 (100)
	Single	3 (23)	0 (0)
**Household income (US $)**			
	<25,000	1 (8)	0 (0)
	25,000**-**49,999	1 (8)	1 (8)
	50,000**-**74,999	1 (8)	2 (15)
	75,000**-**99,999	2 (15)	2 (15)
	100,000**-**149,999	5 (38)	4 (31)
	≥150,000	3 (23)	0 (0)
	Would rather not say	0 (0)	4 (31)
**Highest level of education**			
	Some high school	1 (8)	0 (0)
	High school diploma	0 (0)	0 (0)
	Some postsecondary education	0 (0)	1 (8)
	Postsecondary certificate or diploma	2 (15)	2 (15)
	Postsecondary degree	4 (31)	10 (77)
	Graduate degree	6 (46)	0 (0)
**Number of children**			
	1	6 (46)	2 (15)
	2	4 (31)	3 (23)
	3	1 (8)	8 (62)
	4	2 (15)	0 (0)
**Born in Canada**			
	Yes	8 (62)	1 (8)
	No	5 (38)	12 (92)

### Usability Survey Findings

Overall, parents in both groups found the video helpful, simple to use, and informative. Most French (12/13, 92%) and Filipino (10/13, 77%) parents strongly agreed that the adapted form of the tool was useful. Most parents (10/13, 77% for both French and Filipino parents) also strongly agreed that the tool would help them in making decisions about their child’s health (eg, when to use health services and management of the condition). Table 2 displays the detailed responses to the usability survey of the parents in each group.

**Table 2:** Frequency of responses from French and Filipino parents on usability survey items (N=26).

Items	Strongly agree, n (%)		Agree, n (%)		Not sure, n (%)		Disagree,^a^ n (%)	
	French (n=13)	Filipino (n=13)	French (n=13)	Filipino (n=13)	French (n=13)	Filipino (n=13)	French (n=13)	Filipino (n=13)
It is useful	12 (92)	10 (77)	1 (7)	3 (23)	0 (0)	0 (0)	0 (0)	0 (0)
It provides information that is relevant to me	11 (85)	10 (77)	2 (15)	3 (23)	0 (0)	0 (0)	0 (0)	0 (0)
It is simple to use	9 (69)	10 (77)	1 (7)	3 (23)	3 (23)	0 (0)	0 (0)	0 (0)
I can use it without additional help	10 (77)	6 (46)	1 (7)	6 (46)	1 (7)	1 (7)	1 (7)	0 (0)
Its length is appropriate	10 (77)	10 (77)	2 (15)	2 (15)	1 (7)	1 (7)	0 (0)	0 (0)
It is esthetically pleasing	8 (62)	7 (54)	4 (30)	5 (38)	1 (7)	0 (0)	0 (0)	0 (0)
It helps me to make decisions about my child’s health	10 (77)	10 (77)	3 (23)	3 (23)	0 (0)	0 (0)	0 (0)	0 (0)
I would use it in the future	5 (38)	10 (77)	5 (38)	3 (23)	2 (15)	0 (0)	1 (7)	0 (0)
I would recommend it to a friend	10 (77)	11 (85)	2 (15)	2 (15)	1 (7)	0 (0)	0 (0)	0 (0)

^a^Options were listed on a 5-point Likert scale ranging from *strongly agree* to *strongly disagree*. The *strongly disagree* option was not chosen by any participant in any category.

### Qualitative Interview Findings

#### Overview

All interviews were one**-**on**-**one, with the exception of a Filipino parent dyad who were interviewed together. The analysis of the translated interview transcripts identified the following three major themes: enhancing understanding, relatability, and accessibility. Table 3 displays detailed examples of themes, subthemes, and related participant quotes. It should be noted that the analysis occurred for each community group separately; between-group differences are explained in more detail throughout this section.

**Table 3:** Themes, subthemes, and quotes developed from interviews with French and Filipino parents.

Themes and subthemes		Quotes
**Enhancing understanding**		
	Knowing what to do	“It is rather easy to confuse it with a cold, but you notice a dry and hoarse cough, a noisy breathing.” [Interview_004_French] “I'm even excited to show it and share it to my friends.” [Interview_001_Tagalog]
	Video format	“It’s excellent, yes very well done, simple, clear, the visual helps a lot, I just like the white and the black, you know it’s very well done.” [Interview_012_French] “It seems that the drawing is helpful to get a better understanding of the video.” [Interview_013_Tagalog]
**Relatability**		
	Inclusion in Canadian health care	“I’m more bilingual now than I was before, but when I arrived in 2003, my command of English wasn't as good, and I would have certainly appreciated having this kind of video.” [Interview_013_French] “I feel valued. Just having this, I feel valued already.” [Interview_003_Tagalog]
	Other child health concerns	“[my daughter] was diagnosed with pneumonia the last time we went she was diagnosed but they didn't diagnose her with Asthma, they say treating her for Asthma.” [Interview_007_French] “Most of the children have ear infection.” [Interview_009_Tagalog]
	Video images	“If you’d like to add something cultural, I’ll say you can do it and I’ll add something, a little bit of colors or something that symbols but other than that I think it’s fine.” [Interview_010_French] “It seems that the drawing is helpful to get a better understanding of the video.” [Interview_013_Tagalog]
**Accessibility of information**		
	N/A^a^	“I don’t know where but it will be accessible, but if it’s accessible through social networks, through Youtube there are so many videos, who does anything.” [Interview_006_French] “Usually, the method of delivery is when you go to the hospital or your physician doctor, you get a pamphlet, like that. So this is something that [is] a little bit different, and easily accessible anywhere and you don’t have to really dig for it.” [Interview_012_Tagalog]

^a^N/A: not applicable.

#### Theme 1: Enhancing Understanding

##### Subtheme 1: Knowing What to Do

The main goal of the KT tool presented in this study was to help parents understand how to respond when their child has symptoms consistent with croup. The applicability of the tool to French and Tagalog parents could be assessed through the parents’ gained understanding of the health condition. Participants from both communities commented that they felt more equipped to respond to future occasions on which their children could have croup. It should be mentioned that although none of the parents had experience with their child having croup, several parents did have experience with their child having laryngitis or asthma. One French-speaking parent described noticing their child had “the barking coughs, with the difficulty to breathe”**[**Interview_002_French**]**, so they felt prepared to handle a similar symptom in the future. Parents also voiced their appreciation for the health information that they could pass along to friends and family. A Tagalog-speaking participant said the following:

I’m even excited to show it and share it to my friends.

Interview_001_Tagalog

In both the French and Tagalog translated videos, participants were confused by some of the terminology used. The names of drugs and symptom descriptions were the most commonly confused terms in the video, which were terms without verbatim translations. Parents suggested using sounds and visuals to describe symptoms more clearly. Parents also suggested displaying English words for drug names and health conditions to assist the viewer in future conversations with English-speaking health care providers.

##### Subtheme 2: Video Format

Although parents were presented with a whiteboard animation video for discussion, they were also asked to comment on their preferences regarding the format of KT tools in general. Throughout the interviews, French- and Tagalog-speaking parents described their preferred format as succinct informational videos in simple language. A Tagalog-speaking participant described their positive opinion of the whiteboard animation style in saying the following:

It seems that the drawing is helpful to get a better understanding of the video.

Interview_013_Tagalog

#### Theme 2: Relatability

##### Subtheme 1: Inclusion in Canadian Health Care

Overall, the interviewed French- and Tagalog-speaking parents were pleased with the opportunity to comment on the cultural relevance of the video. Parents liked seeing their languages represented despite most having a high English language proficiency. A Tagalog-speaking parent voiced this appreciation by saying the following:

I feel valued. Just having this, I feel valued already.

Interview_003_Tagalog

##### Subtheme 2: Other Child Health Concerns

Along with commenting on the present video, French- and Tagalog-speaking parents were asked which child health topics they would like to see represented in future KT efforts. French-speaking parents only mentioned the inclusion of information on asthma and other respiratory conditions. Approximately 15% (2/13) of Tagalog-speaking parents wanted more information tools for eczema, whereas others suggested that juvenile diabetes and treatment of burns would improve their knowledge base.

##### Subtheme 3: Video Images

Several Tagalog-speaking parents believed that the video images were already representative of their experiences, as their culture was assimilated into a Western Canadian lifestyle. A participant said the following:

we are more westernized than any other Asians that’s why to translate the video in Tagalog to give out information with regard to health care and with the Canadian setting it’s really awesome.

Interview_005_Tagalog

Others viewing the Tagalog adaptation suggested the inclusion of images of religious items in the household to represent the importance of Catholicism for many Filipino community members. Both French- and Tagalog-speaking parents commented on showing more diverse family representations. French-speaking parents suggested that showing a father in addition to a mother would be helpful, whereas several Tagalog-speaking parents thought the inclusion of grandparents would more accurately depict their experiences. A French-speaking parent mentioned the practical considerations for diverse representation in saying the following:

...French Canadian, another one from West Africa and then another one from North Africa, and then in fact, heterosexuals or homosexuals, there’s lots of possible combinations, I don’t think in 3 minutes you can put it all together.

Interview_013_French

Of note, several parents from both groups situated their experiences as not wholly representative of everyone in their community.

#### Theme 3: Accessibility of Information

Parents were interested in discussing how to improve the accessibility of information to others in their communities and in Canada in general. Their discussions revolved around the format and dissemination avenues. As previously mentioned, parents strongly preferred using videos as a medium for KT tools. Parents described their previous experiences with finding information as challenging and preferred having easy to access web-based resources. A Tagalog-speaking parent described the importance of web-based tools by saying the following:

Usually, the method of delivery is when you go to the hospital or your physician doctor, you get a pamphlet, like that. So this is something that [is] a little bit different, and easily accessible anywhere and you don’t have to really dig for it.

Interview_012_Tagalog

Parents also offered suggestions for where to find this information in the future. Many recommended social media sites where parents are already active. A French-speaking parent suggested that the tools should be “accessible through social networks, through Youtube” [Interview_006_French]. Regardless of their recommendations for further adaptation, their preferences for web-based tools and videos similar to the shown croup video were noticeable.

## Discussion

### Principal Findings

One of the overall goals of our research program is to understand how best to develop and adapt KT tools that are relevant for culturally and linguistically diverse populations. As a first step to support the understanding of best practices when adapting KT products, we adapted a whiteboard animation video for French and Tagalog speakers and sought the perspectives of parents from the respective groups.

Increasingly diverse populations in Alberta and Canada [18] offer new lived experiences and perspectives through which health information can be understood. Efforts to culturally adapt health promotion campaigns [30,31], health care screening inventories [32], and health interventions [33,34] have previously been reported. However, to date, no substantive guiding protocols exist for culturally adapting KT products. As researchers become more interested and able to adapt their KT products to diverse audiences, general considerations will provide guidance in their adaptation endeavors.

By involving stakeholders and engaging end users, we were able to learn some key considerations when adapting tools, which could prove helpful in future KT development or adaptation work. Participants emphasized the importance of enhancing understanding through relatable and accessible KT tool adaptations. French and Filipino community members suggested that future tools should have translations available; primarily use video format; use visuals representative of the community they serve; and be disseminated through commonly used platforms to improve the understanding, relatability, and accessibility of KT tools. On the basis of the recommendations from parents in this study, researchers interested in reaching diverse communities with their KT efforts should consider how best to enhance understanding, relatability, and accessibility within the community of interest. Although these studies involved French and Filipino parents in Alberta, many of the findings could be extended to best practices when engaging with other communities in the KT process. Addressing these areas will undoubtedly look quite different for each specific community of interest; therefore, further patient-centered research should include diverse perspectives.

It is important to acknowledge that culture is not a fixed set of characteristics limited to race and ethnicity; rather, it is a constantly evolving and dynamic concept that encompasses collective views, beliefs, norms, expectations, traditions, customs, and interactions that distinguish population groups [2,3,35]. Therefore, navigating cultural adaptation work can be an involved and complicated process. As mentioned by both Filipino and French parents in this study, understanding the Canadian health care system and Canadian culture more broadly improves with time. Francophone parents were primarily born in Canada; however, Filipino parents who were born elsewhere had an added barrier to accessing Canadian health care services. However, both the French and Filipino parents described that their communities were quite integrated into mainstream Canadian culture; thus, they felt represented by images developed for most cultures. When adapting or developing knowledge products, it is important to assess how the target community has integrated into mainstream Canadian culture. It is likely that newcomers to Canada would experience unique and difficult cultural barriers to accessing health services. Further research into newcomer perspectives in seeking care and how cultural assimilation may affect health information**–**sharing efforts would add value to the field of KT.

Previous efforts to culturally adapt interventions and health promotion materials suggest that engaging with stakeholders in specific communities will produce more impactful tools for knowledge users [31,32,36]. Although very little data are available on how this is implemented in evidence-based KT tools, public health messaging campaigns have explored the cultural nuances that influence knowledge uptake and interpretation [16,30]. There are a few examples of cultural and linguistic adaptations of health promotion materials for specific cultural communities available [31,36]. Telenta et al [31] found that community member engagement allowed for the successful adaptation of a public health campaign for African migrants in Australia. In addition, Bronheim et al [36] further stressed the importance of involving key community partners to assure cultural and linguistic appropriateness in health campaign development and adaptation. Through these examples, researchers emphasize the importance of engaging with specific cultural groups during the creation of health promotion materials [17,31,36] and acknowledging information preferences to ensure effective KT efforts. Despite the recommendation for co-designed KT products, there is little clarity on the protocols for cultural adaptation co-design research.

When adapting or developing KT products for a particular community, there will undoubtedly be unique considerations specific to that cultural group; however, common threads for researcher practice may exist to guide future efforts. Exploring the effective processes that researchers have used during their cultural adaptation work may provide guidance to streamline the adaptation process for others. In addition, crucial for KT tool development is researchers’ willingness to understand the needs of the target community to produce effective tools for health evidence dissemination.

Adapting and translating KT products is a time-consuming and costly endeavor for researchers and may not be achievable for all groups. Certain components of the adaptation process may be prioritized by evaluating the feasibility and impact for the target community. By sharing cultural and linguistic translation processes, we hope to provide guidance for future research efforts in this area.

### Limitations and Future Directions

We acknowledge that this study involved participants from only 2 cultural communities in Alberta, Canada; thus, the findings may not be generalizable to the experiences of those from other cultural groups or in other settings and countries. However, as mentioned by the participants in this study, French and Filipino people’s experiences differ, particularly in terms of access to support. This suggests that cross-cultural adaptation needs may transcend language and culture and be related to how different community groups assimilate, including their awareness of health care support and how to access them. Therefore, understanding the needs of a community and the intricacies of culture may not be fully available to those outside the community.

The process of adapting KT tools for culturally and linguistically diverse communities can be time consuming and involved. Therefore, studies exploring what elements are cross-cutting versus unique to different communities, as well as what elements are related to culture specifically versus familiarity with the local language and system, are warranted.

In addition, future research understanding the needs of other cultural communities using the engagement of families and community leaders and in collaboration with health care providers would add to this nuanced field of KT.

### Conclusions

French- and Tagalog-speaking parents were supportive of the use of English KT products with westernized images but suggested considering cultural components when adapting or developing KT tools. Producing tools with high-quality language translations, video formats, and appropriate and diverse visuals and disseminating on the web would improve understanding, relatability, and accessibility for French- and Tagalog-speaking parents.

As researchers increase their KT efforts, there is a need for more patient-centered cultural adaptation research to inform the development of relevant tools for diverse communities. Important factors such as the community’s integration into mainstream culture, literacy, and newcomer status may influence the adaptation process.

## Appendix

Multimedia Appendix 1 Adapted knowledge translation tools.

Multimedia Appendix 2 Usability survey.

Multimedia Appendix 3 Semistructured interview guide.

## Abbreviations

KT: knowledge translation
