# Supplementary material for: Perspectives From French and Filipino Parents on the Adaptation of Child Health Knowledge Translation Tools: Qualitative Exploration
Source: JMIR Form Res. 2022 Mar 25;6(3):e33156. doi: 10.2196/33156 (PMC8994152; doi:10.2196/33156)
Supplement: Multimedia Appendix 1 [file formative_v6i3e33156_app1.docx]

## **Multimedia Appendix 1.** Adapted knowledge translation tools.

| 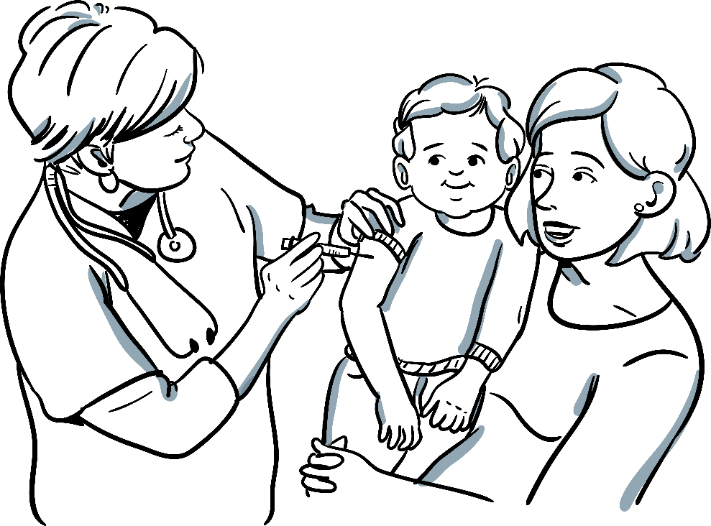  **Original image from the English croup video** | 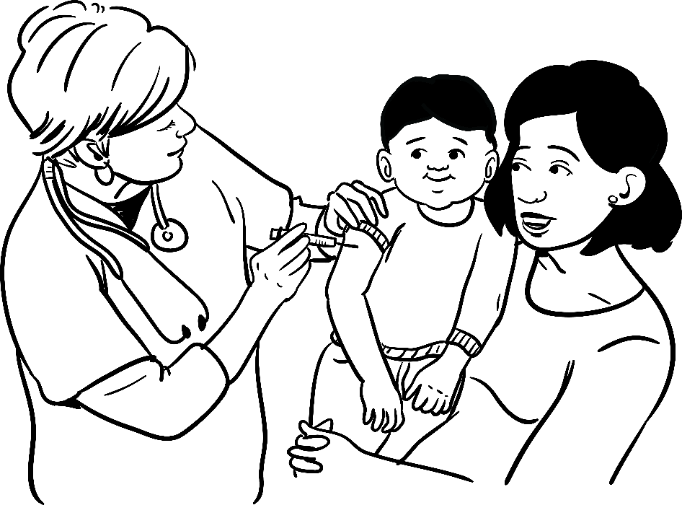**Image from the croup video visually adapted for Tagalog-speaking parents. The design team worked with stakeholder feedback to change the hair color and facial features of the characters.** |
| --- | --- |
| **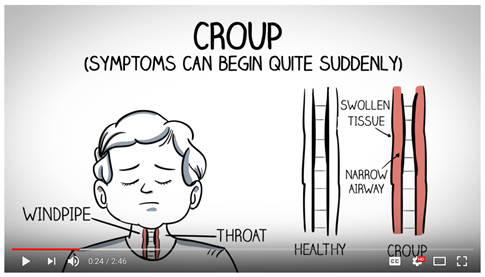**  **Original image from the English croup video** | 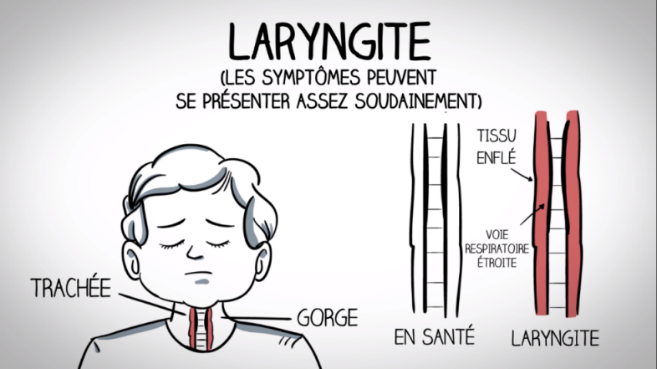  **Image from the croup video linguistically adapted for French-speaking parents.** |
