# Supplementary material for: Perspectives From French and Filipino Parents on the Adaptation of Child Health Knowledge Translation Tools: Qualitative Exploration
Source: JMIR Form Res. 2022 Mar 25;6(3):e33156. doi: 10.2196/33156 (PMC8994152; doi:10.2196/33156)
Supplement: Multimedia Appendix 2 [file formative_v6i3e33156_app2.docx]

**Multimedia Appendix 2.** Usability survey.

SECTION 1: Demographics

1) What is your parenting role?

□ Mother

□ Father

□ Grandmother

□ Grandfather

□ Other:

2) What is your Age?

□ Less than 20 years old

□ 20-30 years

□ 31-40 years

□ 41-50 years

□ 51 years and older

3) What is your Marital Status?

□ Married

□ Single

4) What is your gross annual household income?

□ Less than $25,000

□ $25,000-$49,999

□ $50,000-$74,999

□ $75,000-$99,999

□ $100,000-$149,999

□ $150,000 and over

□ Prefer not to answer

5) What is your highest level of education?

□ Some high school

□ High school diploma

□ Some post-secondary

□ Post-secondary certificate/diploma

□ Post-secondary degree

□ Graduate degree

□ Other

6) How many children do you have? _______

7) How old are your children? _______________

8) Were you born in Canada

□ Yes

□ No

9) a) If no, where were you born?__________________________

b) What year did you move to Canada? ___________________

c) What language(s) do you speak in your home?___________

SECTION 2: Assessment of attributes of the arts-based, digital tools

1. It is useful. [5-point Likert Scale]

2. It provides information that is relevant to me as a parent. [5-point Likert Scale]

3. It is simple to use. [5-point Likert Scale]

4. I can use it without written instructions or additional help. [5-point Likert Scale]

5. Its length is appropriate. [5-point Likert Scale]

6. It is aesthetically pleasing (i.e., images, colours, etc.). [5-point Likert Scale]

7. It helps me to make decisions about my child’s health. [5-point Likert Scale]

8. I would use it in the future. [5-point Likert Scale]

9. I would recommend it to a friend. [5-point Likert Scale]

10. List the most negative aspects: [open text]

11. List the most positive aspects: [open text]
