# Supplementary material for: Perspectives From French and Filipino Parents on the Adaptation of Child Health Knowledge Translation Tools: Qualitative Exploration
Source: JMIR Form Res. 2022 Mar 25;6(3):e33156. doi: 10.2196/33156 (PMC8994152; doi:10.2196/33156)
Supplement: Multimedia Appendix 3 [file formative_v6i3e33156_app3.docx]

**Multimedia Appendix 3.** Semistructured interview guide.

**Linguistic Adaptation**

1. Are there any language features that make understanding the text difficult? (eg, long sentences, difficult grammar, etc.)
2. Is there unfamiliar vocabulary or unclear terminology?
3. Are there any strange or unfamiliar expressions used?
4. Are there any spelling mistakes or other minor problems?

**Cultural Sensitivity/Adaptation**

1. Was it helpful to receive this information in your own language?
2. Are the characters in the video relatable? (i.e Can you identify with any of the characters in the video?)
   - If yes, what aspects of the video make it relatable?
   - If not, why not?
3. How do you think we could make it more relatable?
   - Do you think having culturally relevant images throughout the video are needed?
   - Do you think having socially or culturally relevant themes throughout the video would make it more relatable?
4. Do you think the video is free from any stereotypical images and information?
5. How could the cultural adaptation of this video into French be improved?

**On KT Tools:**

Is the video a useful way to receive health information?

Where would you normally look for health information?
